# The Role of the Evans Index and the Maximal Width of the Frontal Horns of the Lateral Ventricles in the Diagnostic Imaging of Progressive Supranuclear Palsy and Multiple-System Atrophy

**DOI:** 10.3390/diagnostics13162711

**Published:** 2023-08-20

**Authors:** Michał Kutyłowski, Piotr Alster, Natalia Madetko-Alster, Anna Marta Migda, Leszek Królicki, Bartosz Migda

**Affiliations:** 1 Department of Radiology, Mazovian Brodnowski Hospital, 03-242 Warsaw, Poland; 2 Department of Neurology, Medical University of Warsaw, 03-242 Warsaw, Poland; piotr.alster@wum.edu.pl (P.A.); natalia.madetko@wum.edu.pl (N.M.-A.); 3 Department of Internal Medicine and Endocrinology, Medical University of Warsaw, 03-242 Warsaw, Poland; anna.migda@wum.edu.pl; 4 Department of Nuclear Medicine, Mazovian Brodnowski Hospital, 03-242 Warsaw, Poland; leszek.krolicki@wum.edu.pl; 5 Department of Nuclear Medicine, Medical University of Warsaw, 02-097 Warsaw, Poland; 6 Diagnostic Ultrasound Lab., Department of Pediatric Radiology, Medical Faculty, Medical University of Warsaw, 03-242 Warsaw, Poland; bartosz.migda@wum.edu.pl

**Keywords:** atypical parkinsonism, progressive supranuclear palsy, multiple-system atrophy, MR, neuroimaging

## Abstract

Progressive Supranuclear Palsy and Multiple-System Atrophy are entities within the spectrum of atypical parkinsonism. The role of imaging methods in the diagnosis and differentiation between PSP and MSA is limited and Magnetic Resonance Imaging (MRI) is currently used as a reference modality. In this study, the authors examined a group of patients with atypical parkinsonism using a 1.5 T MRI system and aimed to find simple and repeatable measurements that may be useful to distinguish between these diseases. The results of the study indicate that the maximal width of the frontal horns of the lateral ventricles and Evans’ Index may, to some extent, be useful as basic and simple measurements in the diagnostic imaging of patients with atypical parkinsonism.

## 1. Examination of Atypical Parkinsonisms in Clinical Practice and Their Diagnostic Imaging

Progressive Supranuclear Palsy (PSP) and Multiple-System Atrophy (MSA) are atypical parkinsonisms with a relatively poor prognosis after the diagnosis. The duration of the disease varies depending on the subtype; however, it rarely exceeds more than 10 years [1]. The most beneficial prognosis is related to Progressive Supranuclear Palsy—Parkinsonism Predominant [1]. Due to the resemblance of clinical manifestations of MSA—Parkinsonian type (MSA-P) and PSP—Parkinsonism Predominant (PSP-P) related to features as a preservation of cognitive abilities, their slow decline or the presence of dysautonomia, the examination of the entities may be affected by obstacles. Patients with MSA-P or PSP-P have a manifestation with moderate response to levodopa treatment in the early stages and are relatively often misdiagnosed with Parkinson’s Disease (PD). The main limitation in the examination of MSA and PSP is the deficiency of optimal tools in their assessment. In the context of PSP, it is also related to various underlying pathologies among which Corticobasal Degeneration can also be mentioned [2]. Contemporarily, the only method providing a definite diagnosis is possible in a post-mortem examination [2,3]. Among supplementary methods of examination possibly facilitating differential diagnoses in early stages, the most common is neuroimaging. Transcranial sonography, due to its diverse pictures, does not provide sufficient differentiating data in the evaluation of atypical parkinsonisms [1]. Growing interest is associated with nuclear medicine methods with the use of radiotracers such as those highlighting tau, e.g., AV-1451 or PI2620 [4,5]. The methods are affected by a low accessibility, a high cost and off-binding properties. 

Computed Tomography (CT), due to its lower cost and accessibility compared to nuclear medicine methods, and Magnetic Resonance Imaging (MRI) are often used as a first choice modality in the evaluation of brain degeneration; nevertheless, its inferior resolution and commonly occurring artifacts restrict its utilization in more detailed evaluation. 

Magnetic Resonance Imaging is presently regarded as a reference method in the diagnostic imaging of patients with atypical parkinsonism. Though features such as the hot cross bun sign and hummingbird sign are commonly associated with MSA and PSP, their specificity is limited; moreover, they may be described in other entities such as Spinocerebellar Ataxia type 2 or Corticobasal Syndrome [6]. A few useful methods of distinguishing between PSP and MSA were already invented, such as using the mesencephalon-to-pons ratio (M/P), Magnetic Resonance Parkinsonism Index (MRPI) [7] or its expanded version, MRPI 2.0 [8,9], but the patterns they include are rather complex and thus they may be of limited application in ordinary clinical practice. This leads to the necessity for searching for other methods or re-evaluating the currently accessible ones. Having regard to the above, the authors concentrated on an investigation for a single and manageable quantification that may be applied as a simplified differentiating factor between MSA and PSP in MRI. After the analysis, we propose Evans’ Index [10] and the maximum width of the frontal horns of the lateral ventricles as potentially useful measurements. Evans’ Index, which was originally introduced in 1942 and used a pneumoencephalogram as a tool to diagnose hydrocephalus in children [11], after the invention of CT and MRI, has been adapted to these modalities and remains a basic parameter to evaluate the enlargement of the ventricular system with a commonly used value of EI ≥ 0.30 as an indication of ventriculomegaly [12].

## 2. Materials and Methods

The authors included 27 patients in the study (12 males with PSP, 2 males with MSA, 7 females with PSP and 6 females with MSA) with a mean age of 71.9 years for males and 70.4 years for females. All the patients had a diagnosis of PSP or MSA based on the most contemporary criteria [2,3] and did not have any clinically significant comorbidities, such as neoplasms or severe T2-hyperintense white matter lesions—FAZEKAS grade 3 [13], apart from epilepsy, headaches and hypertension. The duration of the symptoms varied from 3 to 6 years. 

All the MRI examinations of the participants of the study were conducted in the Department of Radiology at Mazovian Brodno Hospital using a 1.5 T General Electric Magnetic Resonance system (Chicago, IL, USA) between January 2017 and May 2018 and assessed using dedicated software by a radiologist with experience in neuroimaging.

**Figure 1:**
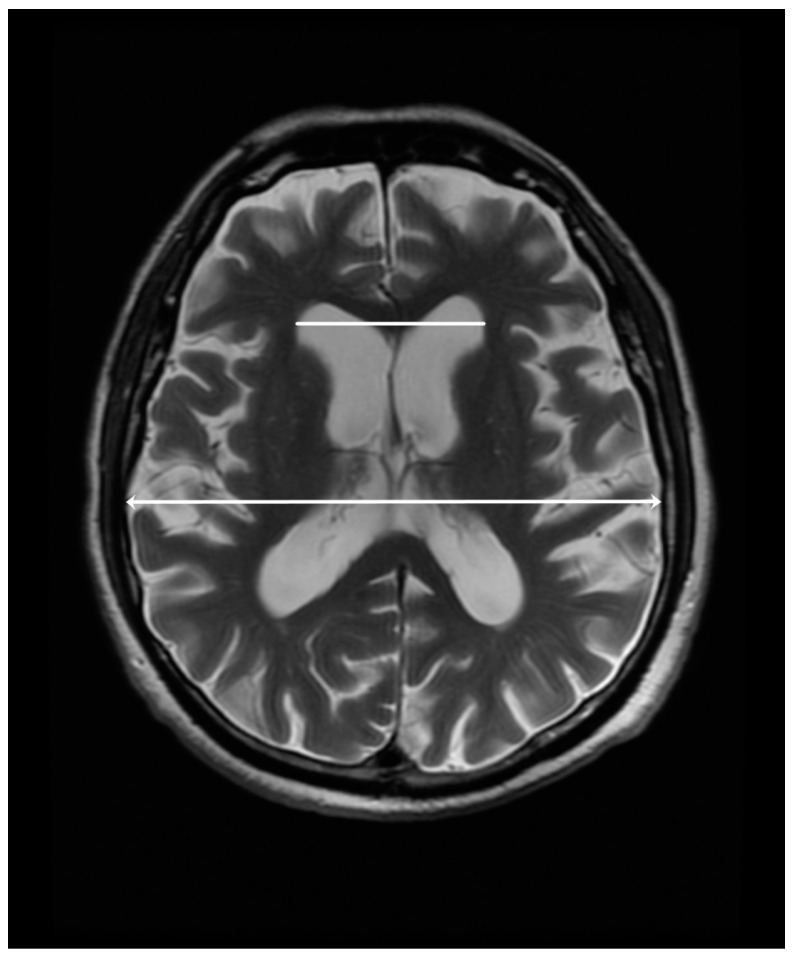
The measurement of the maximal right-to-left width of the frontal horns of the lateral ventricles (48 mm, white line) and the maximal inner diameter of the skull (137 mm, white line with arrowheads) in a 77-year-old male with a clinical diagnosis of PSP. The Evans Index is 0.35 for this patient.

The measurement of the maximum width of the frontal horns (FH) and Evans’ Index (EI) was taken in the axial images parallel to the anterior-commisure–posterior-commisure plane in T2-weighted sequences. We calculated the Evans Index by dividing the maximal right-to-left width of the frontal horns of the lateral ventricles by the maximal inner diameter of the skull at the same level. Figure 1 illustrates the method of measurement of the above-mentioned parameters.

## 3. Statistical Analysis

All statistical analyses were performed with Statistica software (version 13.1. Dell. Inc. Statsoft). Collected data were analyzed according to the type of parameter scales. Normal distribution was proven with the Shapiro–Wilk W test. Subgroup comparison was based on the Student t test due to normal data distribution. For the assessment of variance equality, Leven’s test was used. In the table with descriptive statistics, we provided mean values with minimal and maximal values, standard deviation and its 95% confidence interval. ROC curves were used for the final assessment of significant parameters. In the table, all of the following results are given: the AUC with its confidence interval, and the cut-off point (calculated using the Youden index) with values regarding the sensitivity, specificity, positive predictive value, negative predictive value and accuracy. Finally, an ROC graph for parameter comparison is given. In terms of multiple testing problems, we used Bonferroni correction and a computed *p*-value of 0.025 was used.

## 4. Results

**Figure 2:**
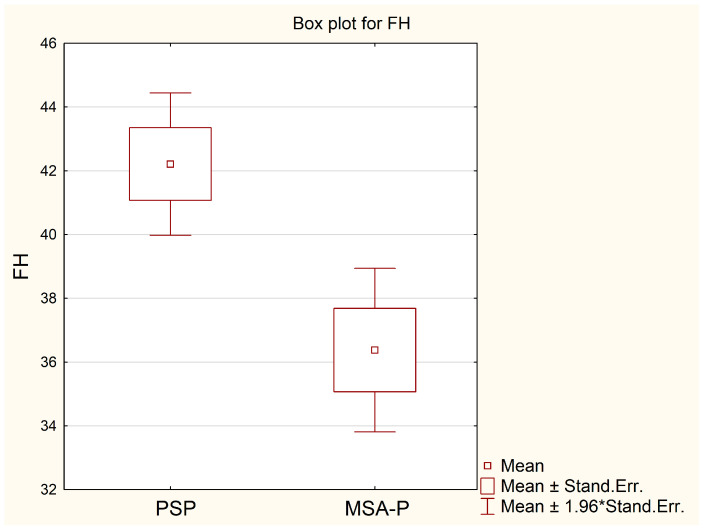
Comparison of mean values of FH in groups of patients with PSP and MSA-P. Stand. Err.—standard error.

**Figure 3:**
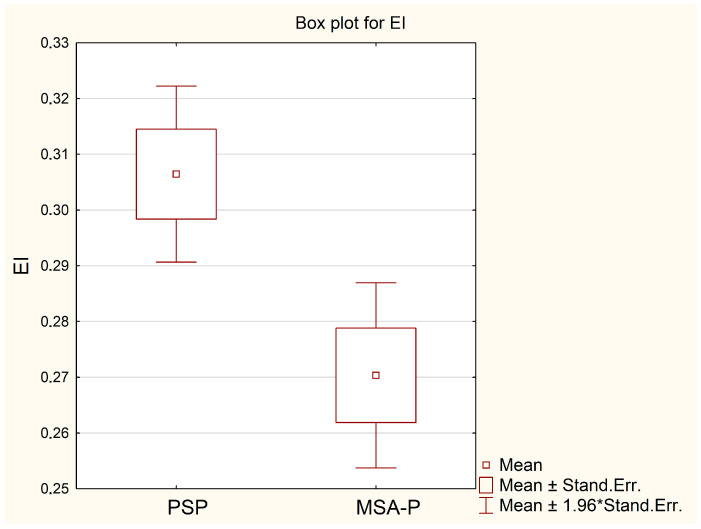
Comparison of mean values of EI in groups of patients with PSP and MSA-P. Stand. Err.—standard error.

**Figure 4:**
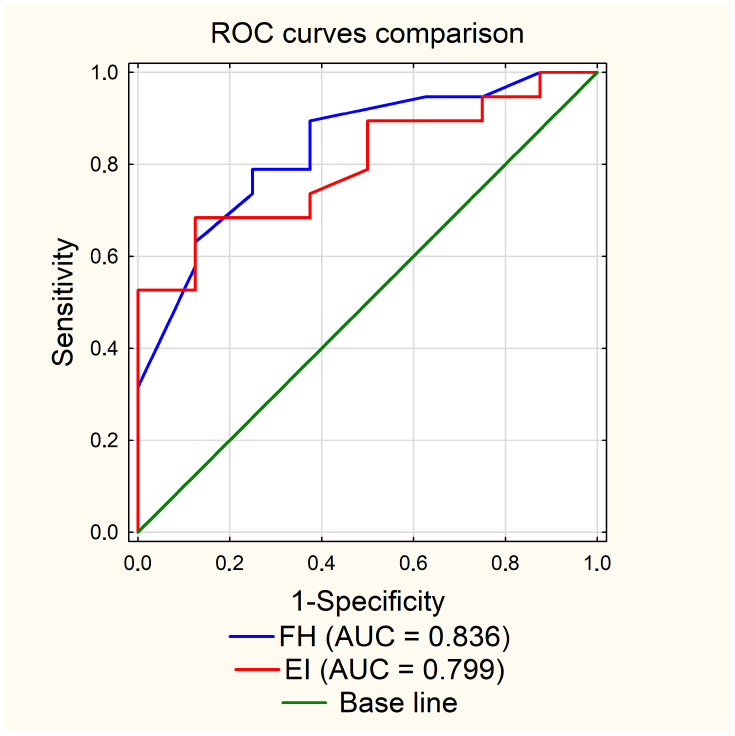
Comparison of ROC curves for FH and EI.

For both analyzed parameters, patients with PSP had higher mean values for the maximal width of frontal horns, 42.2 mm, in relation to patients with MSA-P, 36.4 mm (*p* = 0.0063), as well as for Evans’ Index (EI)—0.306 vs. 0.27 (*p* = 0.0139); see Table 1 and Figure 2 and Figure 3. In the ROC curve analysis, both parameters FH and EI were characterized by high AUC values, 0.836 and 0.799, respectively, in the diagnosis of PSP (Table 2 and Figure 4). The calculated cut-off point for the maximal width of frontal horns was 39 mm. Equal or higher values were related with patients’ diagnosis of progressive supranuclear palsy with a sensitivity of 78.9%, specificity of 75.0%, PPV of 88.2%, NPV of 60.0% and accuracy of 77.8%. An Evans Index equal to 0.292 or higher was also related with PSP patients with a sensitivity of 68.4%, specificity of 87.5%, PPV of 92.9%, NPV of 53.8% and accuracy of 74.1% (Table 2).

## 5. Discussion

The results of the study indicate that both the maximal width of the frontal horns of the lateral ventricles and Evans’ Index may, to some extent, be useful as basic and simple measurements in the diagnostic imaging of patients with atypical parkinsonism. However, it is necessary to underline that, presently, no single nor combined parameter in MRI can be utilized as a decisive factor in differentiation between PSP and MSA, with all the parameters playing a rather supportive role in the diagnosis. The more commonly used parameters such as the M/P ratio [14], MRPI and MRPI 2.0 showed some differences between PSP and MSA [9,15,16,17,18]. All of these measurements are based on the quantification of the midbrain atrophy, which is usually quite sophisticated, time-consuming and requires experience in neuroimaging. The parameters examined in our study show that such basic and easy to conduct calculations as measurements of FH and EI, which do not require any complex software nor automated analyzing systems, may still provide some differential value. Interestingly, among the above-mentioned parameters, only MRPI 2.0 includes the maximum width of the frontal horn in its formula, and regarding our results, an attempt to introduce FH and EI factors into new multi-parametric formulas may be promising. Furthermore, it is to be mentioned that the mean EI in the patients diagnosed with PSP exceeds the commonly used cut-off for the normal value [10], whereas it stays within the normal range in patients with MSA. 

The study is affected by some limitations. The examinations were analyzed by a single radiologist and thus the inter-rater reliability is unknown. All of the diagnoses were based only on the clinical symptoms using the commonly recognized criteria, and as no post-mortem histopathological examinations were conducted, the division between PSP and MSA patients was established on highly probable though not definite diagnoses. The investigated groups of patients are relatively small, which is partly justified with the low prevalence of the entities. Moreover, the group of patients with PSP was not divided into PSP subtypes in order to not reduce the size of the group. Additionally, we used a 1.5 T MRI system, and considering that 3.0 T MRI devices are increasingly available worldwide, the results of the study require confirmation in higher-magnetic-field devices.

## 6. Conclusions

The differentiation between PSP and MSA remains a significant challenge both in clinical and neuroimaging aspects and this study shows that the Evans Index and the maximal width of the frontal horn measurements may play a supportive role in everyday practice as a simple distinguishing factor in differential diagnoses of these entities. Nevertheless, it is to be underlined that, so far, no in vivo diagnostic method nor measurement has been recognized as decisive and enabling to establish an absolutely definite final diagnosis. The major advantage of the proposed factors is their simplicity and repeatability, which may encourage a wider group of practitioners to utilize them. To the best of our knowledge, this is the first work evaluating the usefulness of these measurements in the differentiation between PSP and MSA. A further analysis and possible implementation of these factors into more complex, multiparametric formulas, along with imaginable usage in other diagnostic methods as well, are required.

## Figures and Tables

**Table 1:** Basic statistics.

	PSP (N = 19) (M/F = 12/7)				MSA-P (N = 8) (M/F = 2/6)				*p*
	Mean	Min	Max	SD (95% CI)	Mean	Min	Max	SD (95% CI)	
FH (mm)	42.2	34.0	52.0	5.0 (3.7–7.3)	36.4	30	42	3.7 (2.4–7.5)	0.0063
EI	0.306	0.252	0.38	0.035 (0.027–0.052)	0.27	0.227	0.305	0.024 (0.016–0.049)	0.0139

Legend: SD—standard deviation; CI—confidence interval; *p*—*p* value for Student *t* test.

**Table 2:** ROC curve analysis for PSP diagnosis.

	Cut-off	AUC (95% CI)	Se	Sp	PPV	NPV	Acc
FH	39	0.836 (0.674–0.997)	78.9	75.0	88.2	60.0	77.8
EI	0.292	0.799 (0.632–0.966)	68.4	87.5	92.9	53.8	74.1

Legend: AUC—area under the ROC curve; CI—confidence interval; Se—sensitivity; Sp—specificity; PPV—positive predictive value; NPV—negative predictive value; Acc—accuracy.

## Data Availability

The data presented in this study are available on request.
